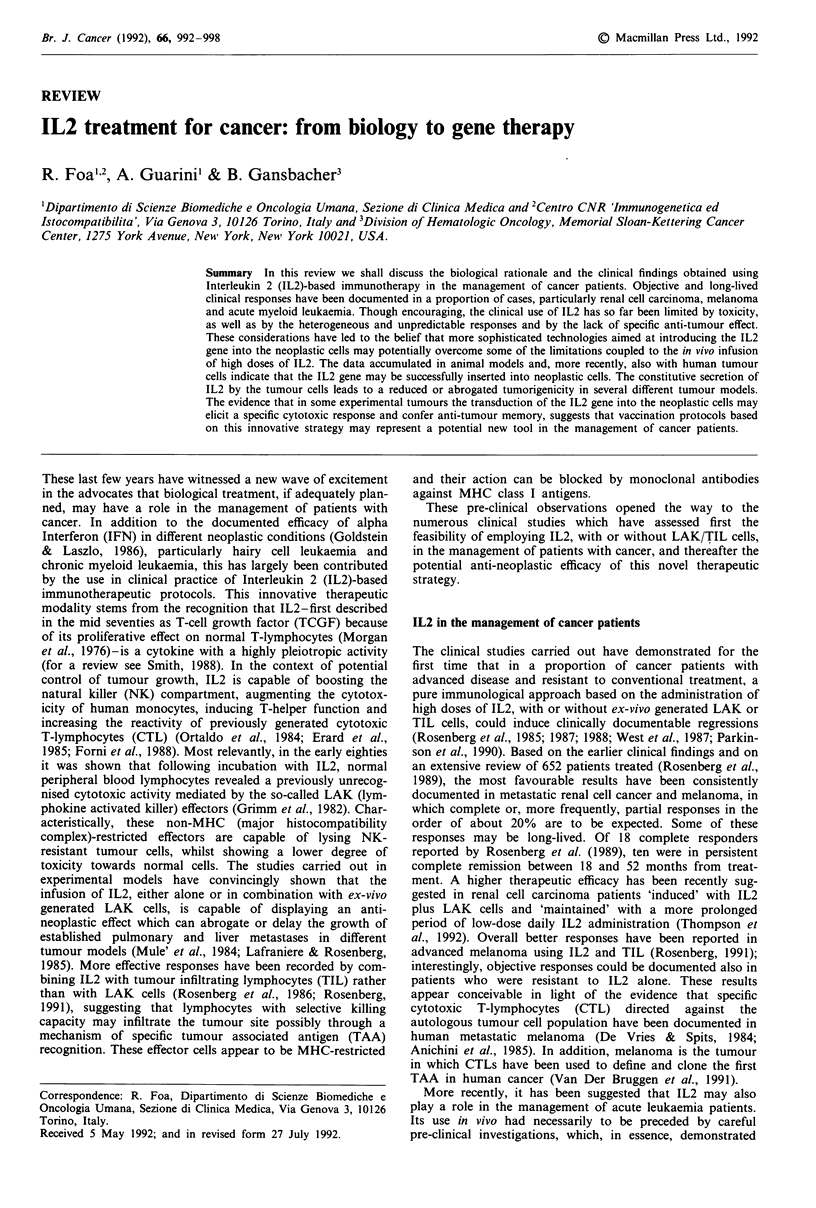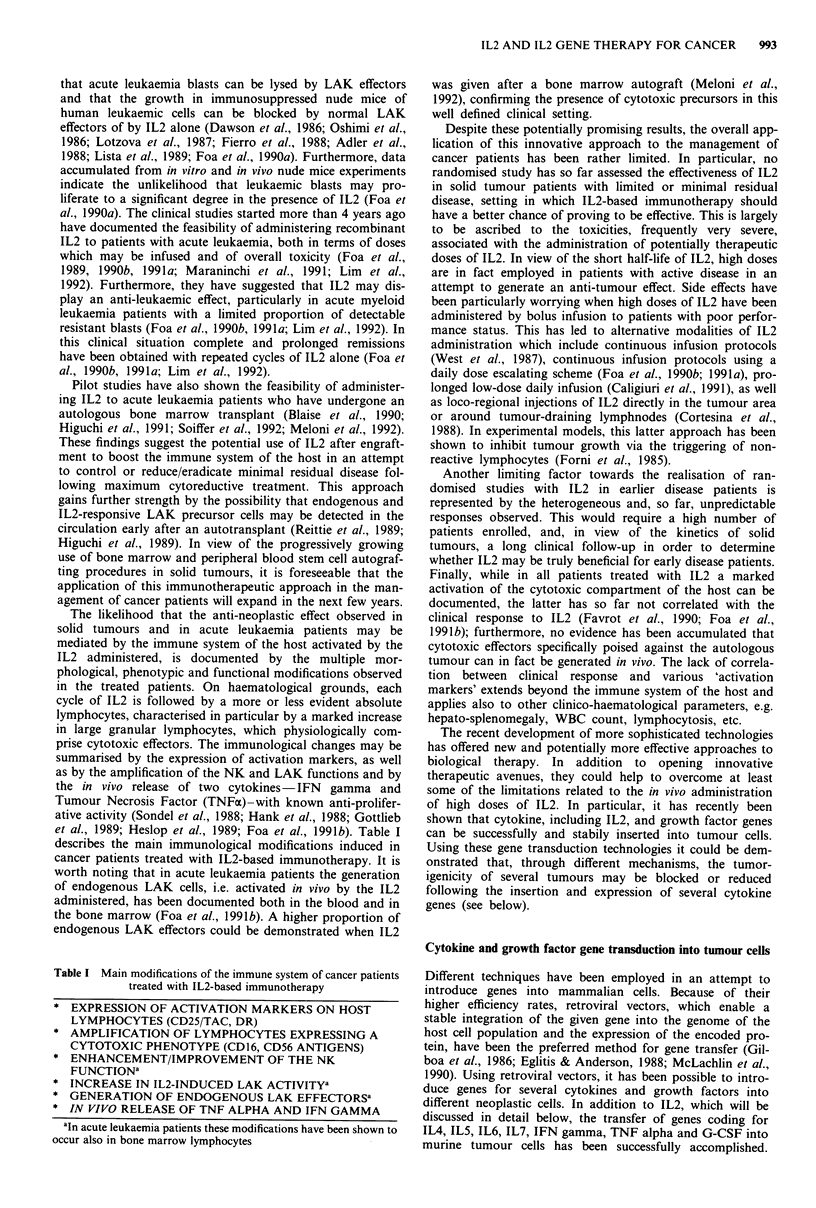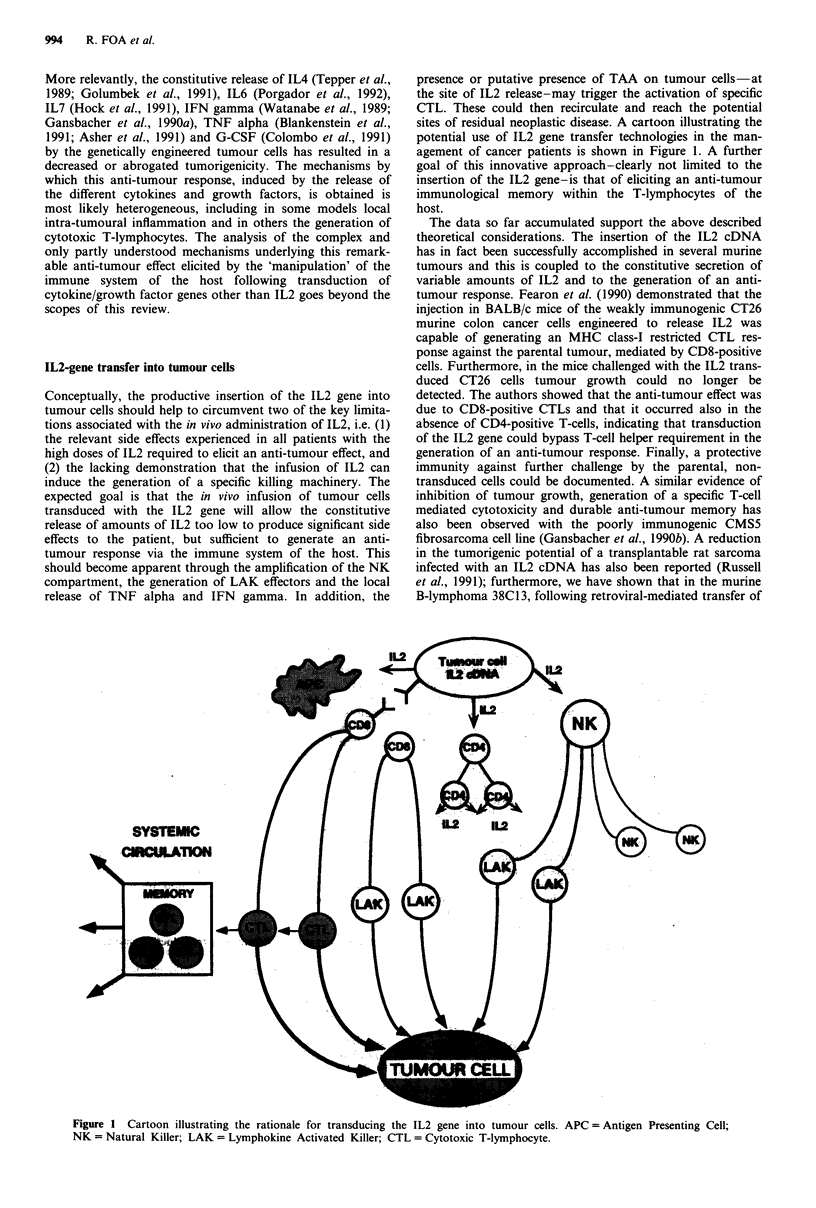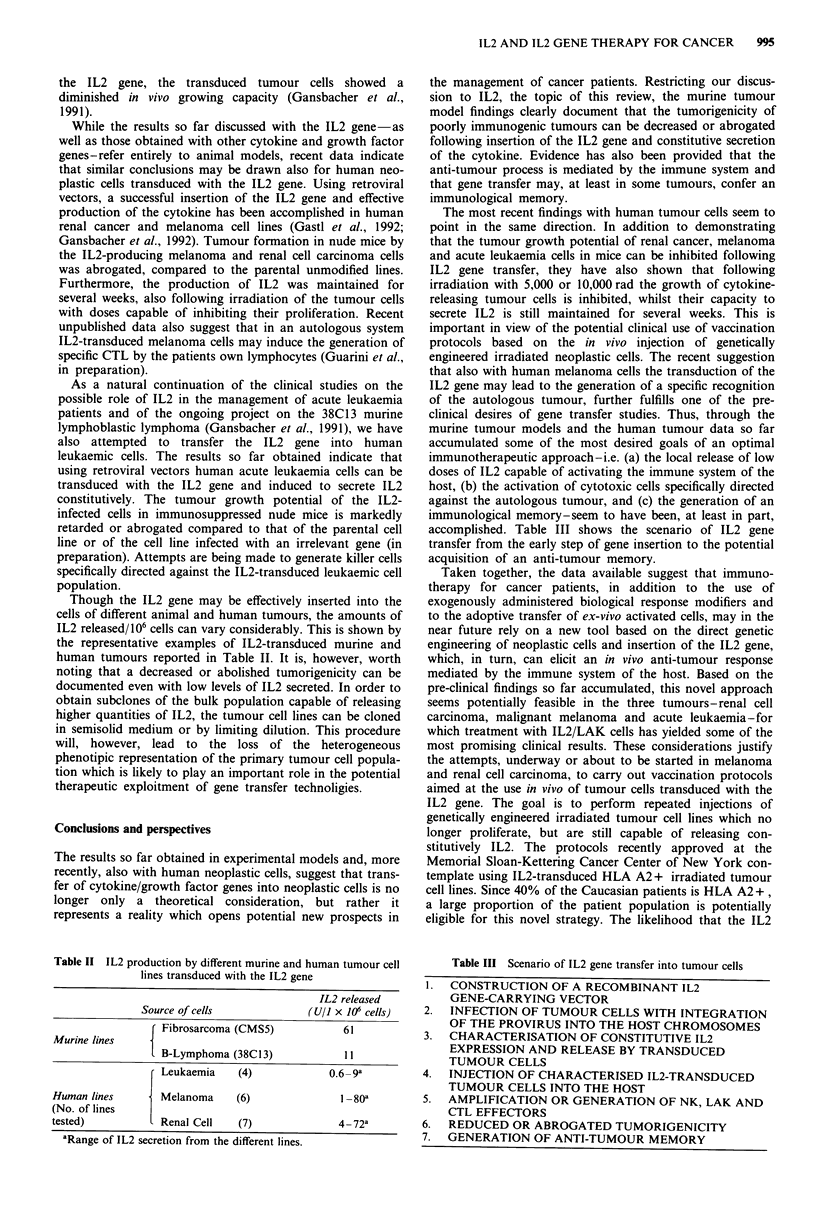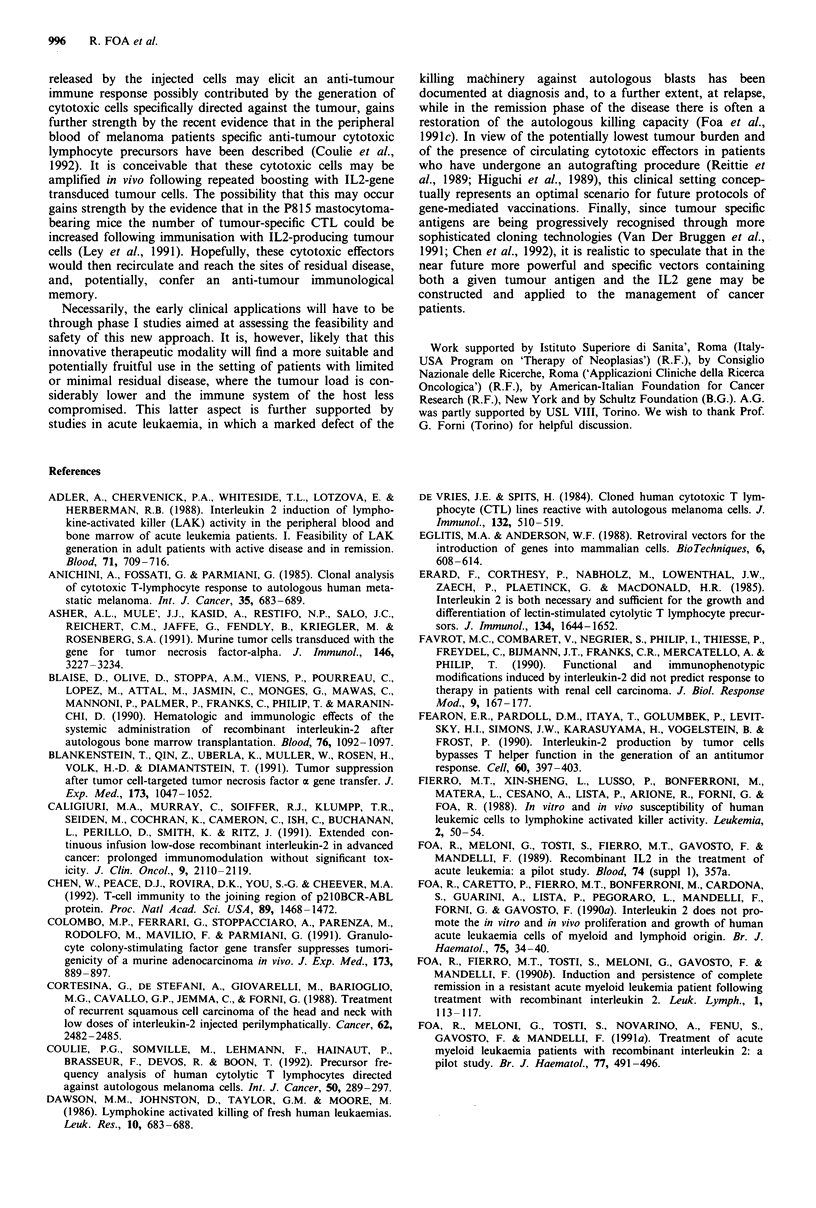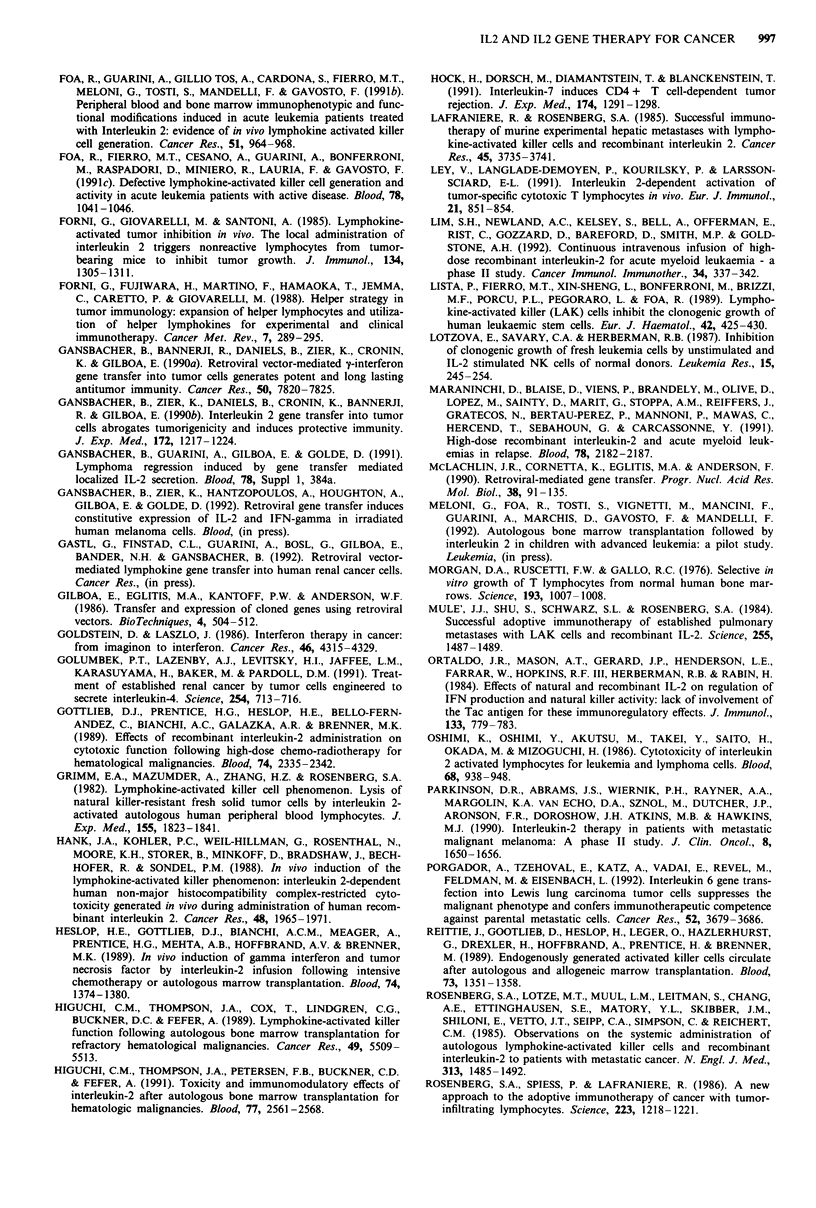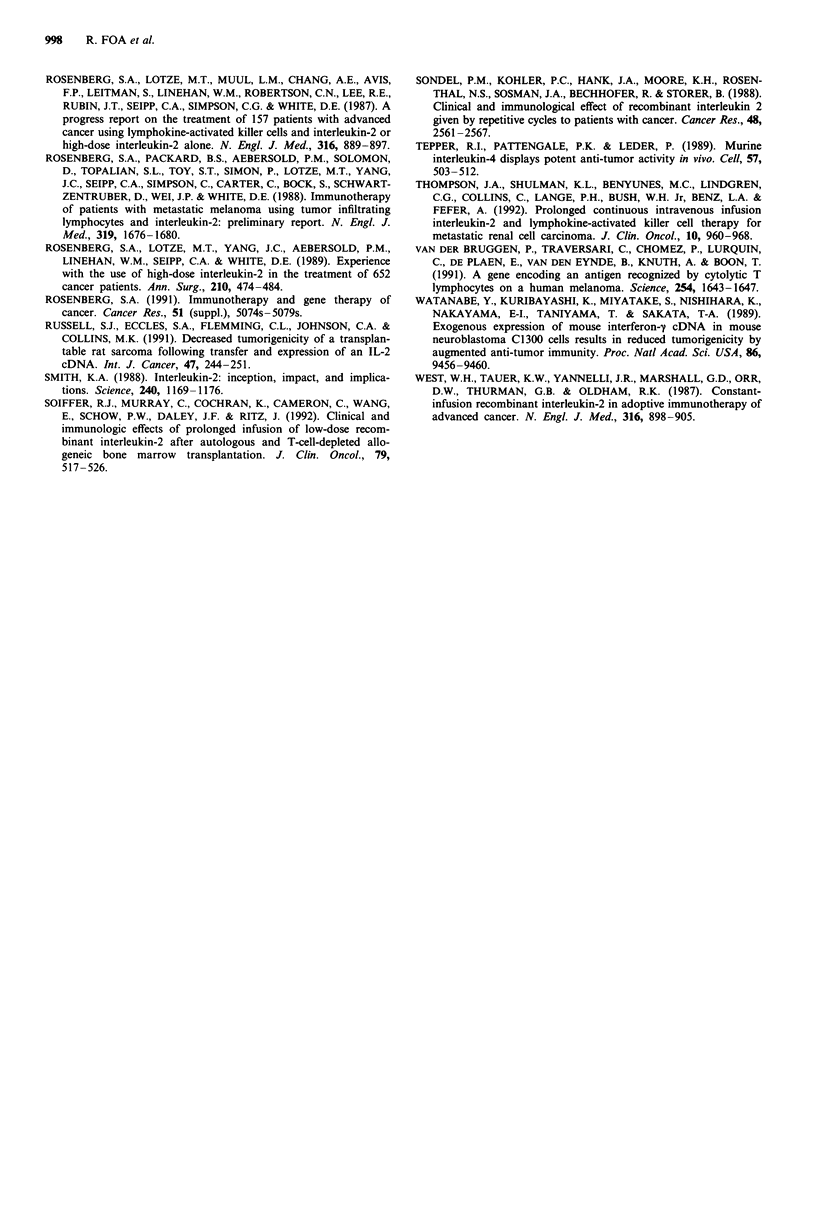# IL2 treatment for cancer: from biology to gene therapy.

**DOI:** 10.1038/bjc.1992.400

**Published:** 1992-12

**Authors:** R. Foa, A. Guarini, B. Gansbacher

**Affiliations:** Dipartimento di Scienze Biomediche e Oncologia Umana, Torino, Italy.

## Abstract

In this review we shall discuss the biological rationale and the clinical findings obtained using Interleukin 2 (IL2)-based immunotherapy in the management of cancer patients. Objective and long-lived clinical responses have been documented in a proportion of cases, particularly renal cell carcinoma, melanoma and acute myeloid leukaemia. Though encouraging, the clinical use of IL2 has so far been limited by toxicity, as well as by the heterogeneous and unpredictable responses and by the lack of specific anti-tumour effect. These considerations have led to the belief that more sophisticated technologies aimed at introducing the IL2 gene into the neoplastic cells may potentially overcome some of the limitations coupled to the in vivo infusion of high doses of IL2. The data accumulated in animal models and, more recently, also with human tumour cells indicate that the IL2 gene may be successfully inserted into neoplastic cells. The constitutive secretion of IL2 by the tumour cells leads to a reduced or abrogated tumorigenicity in several different tumour models. The evidence that in some experimental tumours the transduction of the IL2 gene into the neoplastic cells may elicit a specific cytotoxic response and confer anti-tumour memory, suggests that vaccination protocols based on this innovative strategy may represent a potential new tool in the management of cancer patients.


Br. J. Cancer (1992), 66, 992–998 © Macmillan Press Ltd., 1992

REVIEW

### IL2 treatment for cancer: from biology to gene therapy

R. Foa[1,2], A. Guarini[1] & B. Gansbacher[3]

[1]*Dipartimento di Scienze Biomediche e Oncologia Umana, Sezione di Clinica Medica and* [2]*Centro CNR 'Immunogenetica ed Istocompatibilita', Via Genova 3, 10126 Torino, Italy and* [3]*Division of Hematologic Oncology, Memorial Sloan-Kettering Cancer Center, 1275 York Avenue, New York, New York 10021, USA.*

**Summary** In this review we shall discuss the biological rationale and the clinical findings obtained using Interleukin 2 (IL2)-based immunotherapy in the management of cancer patients. Objective and long-lived clinical responses have been documented in a proportion of cases, particularly renal cell carcinoma, melanoma and acute myeloid leukaemia. Though encouraging, the clinical use of IL2 has so far been limited by toxicity, as well as by the heterogeneous and unpredictable responses and by the lack of specific anti-tumour effect. These considerations have led to the belief that more sophisticated technologies aimed at introducing the IL2 gene into the neoplastic cells may potentially overcome some of the limitations coupled to the *in vivo* infusion of high doses of IL2. The data accumulated in animal models and, more recently, also with human tumour cells indicate that the IL2 gene may be successfully inserted into neoplastic cells. The constitutive secretion of IL2 by the tumour cells leads to a reduced or abrogated tumorigenicity in several different tumour models. The evidence that in some experimental tumours the transduction of the IL2 gene into the neoplastic cells may elicit a specific cytotoxic response and confer anti-tumour memory, suggests that vaccination protocols based on this innovative strategy may represent a potential new tool in the management of cancer patients.

These last few years have witnessed a new wave of excitement in the advocates that biological treatment, if adequately planned, may have a role in the management of patients with cancer. In addition to the documented efficacy of alpha Interferon (IFN) in different neoplastic conditions (Goldstein & Laszlo, 1986), particularly hairy cell leukaemia and chronic myeloid leukaemia, this has largely been contributed by the use in clinical practice of Interleukin 2 (IL2)-based immunotherapeutic protocols. This innovative therapeutic modality stems from the recognition that IL2–first described in the mid seventies as T-cell growth factor (TCGF) because of its proliferative effect on normal T-lymphocytes (Morgan *et al.*, 1976)–is a cytokine with a highly pleiotropic activity (for a review see Smith, 1988). In the context of potential control of tumour growth, IL2 is capable of boosting the natural killer (NK) compartment, augmenting the cytotoxicity of human monocytes, inducing T-helper function and increasing the reactivity of previously generated cytotoxic T-lymphocytes (CTL) (Ortaldo *et al.*, 1984; Erard *et al.*, 1985; Forni *et al.*, 1988). Most relevantly, in the early eighties it was shown that following incubation with IL2, normal peripheral blood lymphocytes revealed a previously unrecognised cytotoxic activity mediated by the so-called LAK (lymphokine activated killer) effectors (Grimm *et al.*, 1982). Characteristically, these non-MHC (major histocompatibility complex)-restricted effectors are capable of lysing NK-resistant tumour cells, whilst showing a lower degree of toxicity towards normal cells. The studies carried out in experimental models have convincingly shown that the infusion of IL2, either alone or in combination with *ex-vivo* generated LAK cells, is capable of displaying an anti-neoplastic effect which can abrogate or delay the growth of established pulmonary and liver metastases in different tumour models (Mule' *et al.*, 1984; Lafraniere & Rosenberg, 1985). More effective responses have been recorded by combining IL2 with tumour infiltrating lymphocytes (TIL) rather than with LAK cells (Rosenberg *et al.*, 1986; Rosenberg, 1991), suggesting that lymphocytes with selective killing capacity may infiltrate the tumour site possibly through a mechanism of specific tumour associated antigen (TAA) recognition. These effector cells appear to be MHC-restricted and their action can be blocked by monoclonal antibodies against MHC class I antigens.

These pre-clinical observations opened the way to the numerous clinical studies which have assessed first the feasibility of employing IL2, with or without LAK/TIL cells, in the management of patients with cancer, and thereafter the potential anti-neoplastic efficacy of this novel therapeutic strategy.

### IL2 in the management of cancer patients

The clinical studies carried out have demonstrated for the first time that in a proportion of cancer patients with advanced disease and resistant to conventional treatment, a pure immunological approach based on the administration of high doses of IL2, with or without *ex-vivo* generated LAK or TIL cells, could induce clinically documentable regressions (Rosenberg *et al.*, 1985; 1987; 1988; West *et al.*, 1987; Parkinson *et al.*, 1990). Based on the earlier clinical findings and on an extensive review of 652 patients treated (Rosenberg *et al.*, 1989), the most favourable results have been consistently documented in metastatic renal cell cancer and melanoma, in which complete or, more frequently, partial responses in the order of about 20% are to be expected. Some of these responses may be long-lived. Of 18 complete responders reported by Rosenberg *et al.* (1989), ten were in persistent complete remission between 18 and 52 months from treatment. A higher therapeutic efficacy has been recently suggested in renal cell carcinoma patients 'induced' with IL2 plus LAK cells and 'maintained' with a more prolonged period of low-dose daily IL2 administration (Thompson *et al.*, 1992). Overall better responses have been reported in advanced melanoma using IL2 and TIL (Rosenberg, 1991); interestingly, objective responses could be documented also in patients who were resistant to IL2 alone. These results appear conceivable in light of the evidence that specific cytotoxic T-lymphocytes (CTL) directed against the autologous tumour cell population have been documented in human metastatic melanoma (De Vries & Spits, 1984; Anichini *et al.*, 1985). In addition, melanoma is the tumour in which CTLs have been used to define and clone the first TAA in human cancer (Van Der Bruggen *et al.*, 1991).

More recently, it has been suggested that IL2 may also play a role in the management of acute leukaemia patients. Its use *in vivo* had necessarily to be preceded by careful pre-clinical investigations, which, in essence, demonstrated

Correspondence: R. Foa, Dipartimento di Scienze Biomediche e Oncologia Umana, Sezione di Clinica Medica, Via Genova 3, 10126 Torino, Italy.
Received 5 May 1992; and in revised form 27 July 1992.

that acute leukaemia blasts can be lysed by LAK effectors and that the growth in immunosuppressed nude mice of human leukaemic cells can be blocked by normal LAK effectors of by IL2 alone (Dawson *et al.*, 1986; Oshimi *et al.*, 1986; Lotzova *et al.*, 1987; Fierro *et al.*, 1988; Adler *et al.*, 1988; Lista *et al.*, 1989; Foa *et al.*, 1990*a*). Furthermore, data accumulated from *in vitro* and *in vivo* nude mice experiments indicate the unlikelihood that leukaemic blasts may proliferate to a significant degree in the presence of IL2 (Foa *et al.*, 1990*a*). The clinical studies started more than 4 years ago have documented the feasibility of administering recombinant IL2 to patients with acute leukaemia, both in terms of doses which may be infused and of overall toxicity (Foa *et al.*, 1989, 1990*b*, 1991*a*; Maraninchi *et al.*, 1991; Lim *et al.*, 1992). Furthermore, they have suggested that IL2 may display an anti-leukaemic effect, particularly in acute myeloid leukaemia patients with a limited proportion of detectable resistant blasts (Foa *et al.*, 1990*b*, 1991*a*; Lim *et al.*, 1992). In this clinical situation complete and prolonged remissions have been obtained with repeated cycles of IL2 alone (Foa *et al.*, 1990*b*, 1991*a*; Lim *et al.*, 1992).

Pilot studies have also shown the feasibility of administering IL2 to acute leukaemia patients who have undergone an autologous bone marrow transplant (Blaise *et al.*, 1990; Higuchi *et al.*, 1991; Soiffer *et al.*, 1992; Meloni *et al.*, 1992). These findings suggest the potential use of IL2 after engraftment to boost the immune system of the host in an attempt to control or reduce/eradicate minimal residual disease following maximum cytoreductive treatment. This approach gains further strength by the possibility that endogenous and IL2-responsive LAK precursor cells may be detected in the circulation early after an autotransplant (Reittie *et al.*, 1989; Higuchi *et al.*, 1989). In view of the progressively growing use of bone marrow and peripheral blood stem cell autografting procedures in solid tumours, it is foreseeable that the application of this immunotherapeutic approach in the management of cancer patients will expand in the next few years.

The likelihood that the anti-neoplastic effect observed in solid tumours and in acute leukaemia patients may be mediated by the immune system of the host activated by the IL2 administered, is documented by the multiple morphological, phenotypic and functional modifications observed in the treated patients. On haematological grounds, each cycle of IL2 is followed by a more or less evident absolute lymphocytes, characterised in particular by a marked increase in large granular lymphocytes, which physiologically comprise cytotoxic effectors. The immunological changes may be summarised by the expression of activation markers, as well as by the amplification of the NK and LAK functions and by the *in vivo* release of two cytokines—IFN gamma and Tumour Necrosis Factor (TNFα)-with known anti-proliferative activity (Sondel *et al.*, 1988; Hank *et al.*, 1988; Gottlieb *et al.*, 1989; Heslop *et al.*, 1989; Foa *et al.*, 1991*b*). Table I describes the main immunological modifications induced in cancer patients treated with IL2-based immunotherapy. It is worth noting that in acute leukaemia patients the generation of endogenous LAK cells, i.e. activated *in vivo* by the IL2 administered, has been documented both in the blood and in the bone marrow (Foa *et al.*, 1991*b*). A higher proportion of endogenous LAK effectors could be demonstrated when IL2 was given after a bone marrow autograft (Meloni *et al.*, 1992), confirming the presence of cytotoxic precursors in this well defined clinical setting.

**Table I** Main modifications of the immune system of cancer patients treated with IL2-based immunotherapy

| |
|---|
| * EXPRESSION OF ACTIVATION MARKERS ON HOST LYMPHOCYTES (CD25/TAC, DR) |
| * AMPLIFICATION OF LYMPHOCYTES EXPRESSING A CYTOTOXIC PHENOTYPE (CD16, CD56 ANTIGENS) |
| * ENHANCEMENT/IMPROVEMENT OF THE NK FUNCTION[a] |
| * INCREASE IN IL2-INDUCED LAK ACTIVITY[a] |
| * GENERATION OF ENDOGENOUS LAK EFFECTORS[a] |
| * *IN VIVO* RELEASE OF TNF ALPHA AND IFN GAMMA |

[a]In acute leukaemia patients these modifications have been shown to occur also in bone marrow lymphocytes

Despite these potentially promising results, the overall application of this innovative approach to the management of cancer patients has been rather limited. In particular, no randomised study has so far assessed the effectiveness of IL2 in solid tumour patients with limited or minimal residual disease, setting in which IL2-based immunotherapy should have a better chance of proving to be effective. This is largely to be ascribed to the toxicities, frequently very severe, associated with the administration of potentially therapeutic doses of IL2. In view of the short half-life of IL2, high doses are in fact employed in patients with active disease in an attempt to generate an anti-tumour effect. Side effects have been particularly worrying when high doses of IL2 have been administered by bolus infusion to patients with poor performance status. This has led to alternative modalities of IL2 administration which include continuous infusion protocols (West *et al.*, 1987), continuous infusion protocols using a daily dose escalating scheme (Foa *et al.*, 1990*b*; 1991*a*), prolonged low-dose daily infusion (Caligiuri *et al.*, 1991), as well as loco-regional injections of IL2 directly in the tumour area or around tumour-draining lymphnodes (Cortesina *et al.*, 1988). In experimental models, this latter approach has been shown to inhibit tumour growth via the triggering of nonreactive lymphocytes (Forni *et al.*, 1985).

Another limiting factor towards the realisation of randomised studies with IL2 in earlier disease patients is represented by the heterogeneous and, so far, unpredictable responses observed. This would require a high number of patients enrolled, and, in view of the kinetics of solid tumours, a long clinical follow-up in order to determine whether IL2 may be truly beneficial for early disease patients. Finally, while in all patients treated with IL2 a marked activation of the cytotoxic compartment of the host can be documented, the latter has so far not correlated with the clinical response to IL2 (Favrot *et al.*, 1990; Foa *et al.*, 1991*b*); furthermore, no evidence has been accumulated that cytotoxic effectors specifically poised against the autologous tumour can in fact be generated *in vivo*. The lack of correlation between clinical response and various 'activation markers' extends beyond the immune system of the host and applies also to other clinico-haematological parameters, e.g. hepato-splenomegaly, WBC count, lymphocytosis, etc.

The recent development of more sophisticated technologies has offered new and potentially more effective approaches to biological therapy. In addition to opening innovative therapeutic avenues, they could help to overcome at least some of the limitations related to the *in vivo* administration of high doses of IL2. In particular, it has recently been shown that cytokine, including IL2, and growth factor genes can be successfully and stabily inserted into tumour cells. Using these gene transduction technologies it could be demonstrated that, through different mechanisms, the tumorigenicity of several tumours may be blocked or reduced following the insertion and expression of several cytokine genes (see below).

### Cytokine and growth factor gene transduction into tumour cells

Different techniques have been employed in an attempt to introduce genes into mammalian cells. Because of their higher efficiency rates, retroviral vectors, which enable a stable integration of the given gene into the genome of the host cell population and the expression of the encoded protein, have been the preferred method for gene transfer (Gilboa *et al.*, 1986; Eglitis & Anderson, 1988; McLachlin *et al.*, 1990). Using retroviral vectors, it has been possible to introduce genes for several cytokines and growth factors into different neoplastic cells. In addition to IL2, which will be discussed in detail below, the transfer of genes coding for IL4, IL5, IL6, IL7, IFN gamma, TNF alpha and G-CSF into murine tumour cells has been successfully accomplished.

More relevantly, the constitutive release of IL4 (Tepper *et al.*, 1989; Golumbek *et al.*, 1991), IL6 (Porgador *et al.*, 1992), IL7 (Hock *et al.*, 1991), IFN gamma (Watanabe *et al.*, 1989; Gansbacher *et al.*, 1990*a*), TNF alpha (Blankenstein *et al.*, 1991; Asher *et al.*, 1991) and G-CSF (Colombo *et al.*, 1991) by the genetically engineered tumour cells has resulted in a decreased or abrogated tumorigenicity. The mechanisms by which this anti-tumour response, induced by the release of the different cytokines and growth factors, is obtained is most likely heterogeneous, including in some models local intra-tumoural inflammation and in others the generation of cytotoxic T-lymphocytes. The analysis of the complex and only partly understood mechanisms underlying this remarkable anti-tumour effect elicited by the 'manipulation' of the immune system of the host following transduction of cytokine/growth factor genes other than IL2 goes beyond the scopes of this review.

### IL2-gene transfer into tumour cells

Conceptually, the productive insertion of the IL2 gene into tumour cells should help to circumvent two of the key limitations associated with the *in vivo* administration of IL2, i.e. (1) the relevant side effects experienced in all patients with the high doses of IL2 required to elicit an anti-tumour effect, and (2) the lacking demonstration that the infusion of IL2 can induce the generation of a specific killing machinery. The expected goal is that the *in vivo* infusion of tumour cells transduced with the IL2 gene will allow the constitutive release of amounts of IL2 too low to produce significant side effects to the patient, but sufficient to generate an anti-tumour response via the immune system of the host. This should become apparent through the amplification of the NK compartment, the generation of LAK effectors and the local release of TNF alpha and IFN gamma. In addition, the presence or putative presence of TAA on tumour cells—at the site of IL2 release-may trigger the activation of specific CTL. These could then recirculate and reach the potential sites of residual neoplastic disease. A cartoon illustrating the potential use of IL2 gene transfer technologies in the management of cancer patients is shown in Figure 1. A further goal of this innovative approach-clearly not limited to the insertion of the IL2 gene-is that of eliciting an anti-tumour immunological memory within the T-lymphocytes of the host.

The data so far accumulated support the above described theoretical considerations. The insertion of the IL2 cDNA has in fact been successfully accomplished in several murine tumours and this is coupled to the constitutive secretion of variable amounts of IL2 and to the generation of an anti-tumour response. Fearon *et al.* (1990) demonstrated that the injection in BALB/c mice of the weakly immunogenic CT26 murine colon cancer cells engineered to release IL2 was capable of generating an MHC class-I restricted CTL response against the parental tumour, mediated by CD8-positive cells. Furthermore, in the mice challenged with the IL2 transduced CT26 cells tumour growth could no longer be detected. The authors showed that the anti-tumour effect was due to CD8-positive CTLs and that it occurred also in the absence of CD4-positive T-cells, indicating that transduction of the IL2 gene could bypass T-cell helper requirement in the generation of an anti-tumour response. Finally, a protective immunity against further challenge by the parental, non-transduced cells could be documented. A similar evidence of inhibition of tumour growth, generation of a specific T-cell mediated cytotoxicity and durable anti-tumour memory has also been observed with the poorly immunogenic CMS5 fibrosarcoma cell line (Gansbacher *et al.*, 1990*b*). A reduction in the tumorigenic potential of a transplantable rat sarcoma infected with an IL2 cDNA has also been reported (Russell *et al.*, 1991); furthermore, we have shown that in the murine B-lymphoma 38C13, following retroviral-mediated transfer of

**Figure 1** Cartoon illustrating the rationale for transducing the IL2 gene into tumour cells. APC = Antigen Presenting Cell; NK = Natural Killer; LAK = Lymphokine Activated Killer; CTL = Cytotoxic T-lymphocyte.

the IL2 gene, the transduced tumour cells showed a diminished *in vivo* growing capacity (Gansbacher *et al.*, 1991).

While the results so far discussed with the IL2 gene—as well as those obtained with other cytokine and growth factor genes–refer entirely to animal models, recent data indicate that similar conclusions may be drawn also for human neoplastic cells transduced with the IL2 gene. Using retroviral vectors, a successful insertion of the IL2 gene and effective production of the cytokine has been accomplished in human renal cancer and melanoma cell lines (Gastl *et al.*, 1992; Gansbacher *et al.*, 1992). Tumour formation in nude mice by the IL2-producing melanoma and renal cell carcinoma cells was abrogated, compared to the parental unmodified lines. Furthermore, the production of IL2 was maintained for several weeks, also following irradiation of the tumour cells with doses capable of inhibiting their proliferation. Recent unpublished data also suggest that in an autologous system IL2-transduced melanoma cells may induce the generation of specific CTL by the patients own lymphocytes (Guarini *et al.*, in preparation).

As a natural continuation of the clinical studies on the possible role of IL2 in the management of acute leukaemia patients and of the ongoing project on the 38C13 murine lymphoblastic lymphoma (Gansbacher *et al.*, 1991), we have also attempted to transfer the IL2 gene into human leukaemic cells. The results so far obtained indicate that using retroviral vectors human acute leukaemia cells can be transduced with the IL2 gene and induced to secrete IL2 constitutively. The tumour growth potential of the IL2-infected cells in immunosuppressed nude mice is markedly retarded or abrogated compared to that of the parental cell line or of the cell line infected with an irrelevant gene (in preparation). Attempts are being made to generate killer cells specifically directed against the IL2-transduced leukaemic cell population.

Though the IL2 gene may be effectively inserted into the cells of different animal and human tumours, the amounts of IL2 released/$10^6$ cells can vary considerably. This is shown by the representative examples of IL2-transduced murine and human tumours reported in Table II. It is, however, worth noting that a decreased or abolished tumorigenicity can be documented even with low levels of IL2 secreted. In order to obtain subclones of the bulk population capable of releasing higher quantities of IL2, the tumour cell lines can be cloned in semisolid medium or by limiting dilution. This procedure will, however, lead to the loss of the heterogeneous phenotipic representation of the primary tumour cell population which is likely to play an important role in the potential therapeutic exploitment of gene transfer technologies.

### Conclusions and perspectives

The results so far obtained in experimental models and, more recently, also with human neoplastic cells, suggest that transfer of cytokine/growth factor genes into neoplastic cells is no longer only a theoretical consideration, but rather it represents a reality which opens potential new prospects in the management of cancer patients. Restricting our discussion to IL2, the topic of this review, the murine tumour model findings clearly document that the tumorigenicity of poorly immunogenic tumours can be decreased or abrogated following insertion of the IL2 gene and constitutive secretion of the cytokine. Evidence has also been provided that the anti-tumour process is mediated by the immune system and that gene transfer may, at least in some tumours, confer an immunological memory.

The most recent findings with human tumour cells seem to point in the same direction. In addition to demonstrating that the tumour growth potential of renal cancer, melanoma and acute leukaemia cells in mice can be inhibited following IL2 gene transfer, they have also shown that following irradiation with 5,000 or 10,000 rad the growth of cytokine-releasing tumour cells is inhibited, whilst their capacity to secrete IL2 is still maintained for several weeks. This is important in view of the potential clinical use of vaccination protocols based on the *in vivo* injection of genetically engineered irradiated neoplastic cells. The recent suggestion that also with human melanoma cells the transduction of the IL2 gene may lead to the generation of a specific recognition of the autologous tumour, further fulfills one of the preclinical desires of gene transfer studies. Thus, through the murine tumour models and the human tumour data so far accumulated some of the most desired goals of an optimal immunotherapeutic approach–i.e. (a) the local release of low doses of IL2 capable of activating the immune system of the host, (b) the activation of cytotoxic cells specifically directed against the autologous tumour, and (c) the generation of an immunological memory–seem to have been, at least in part, accomplished. Table III shows the scenario of IL2 gene transfer from the early step of gene insertion to the potential acquisition of an anti-tumour memory.

Taken together, the data available suggest that immunotherapy for cancer patients, in addition to the use of exogenously administered biological response modifiers and to the adoptive transfer of *ex-vivo* activated cells, may in the near future rely on a new tool based on the direct genetic engineering of neoplastic cells and insertion of the IL2 gene, which, in turn, can elicit an *in vivo* anti-tumour response mediated by the immune system of the host. Based on the pre-clinical findings so far accumulated, this novel approach seems potentially feasible in the three tumours–renal cell carcinoma, malignant melanoma and acute leukaemia–for which treatment with IL2/LAK cells has yielded some of the most promising clinical results. These considerations justify the attempts, underway or about to be started in melanoma and renal cell carcinoma, to carry out vaccination protocols aimed at the use *in vivo* of tumour cells transduced with the IL2 gene. The goal is to perform repeated injections of genetically engineered irradiated tumour cell lines which no longer proliferate, but are still capable of releasing constitutively IL2. The protocols recently approved at the Memorial Sloan-Kettering Cancer Center of New York contemplate using IL2-transduced HLA A2+ irradiated tumour cell lines. Since 40% of the Caucasian patients is HLA A2+, a large proportion of the patient population is potentially eligible for this novel strategy. The likelihood that the IL2

**Table II** IL2 production by different murine and human tumour cell lines transduced with the IL2 gene

| | *Source of cells* | | *IL2 released (U/1 × $10^6$ cells)* |
|---|---|---|---|
| *Murine lines* | Fibrosarcoma (CMS5) | | 61 |
| | B-Lymphoma (38C13) | | 11 |
| *Human lines* (No. of lines tested) | Leukaemia | (4) | 0.6–9[a] |
| | Melanoma | (6) | 1–80[a] |
| | Renal Cell | (7) | 4–72[a] |

[a]Range of IL2 secretion from the different lines.

**Table III** Scenario of IL2 gene transfer into tumour cells

1. CONSTRUCTION OF A RECOMBINANT IL2 GENE-CARRYING VECTOR
2. INFECTION OF TUMOUR CELLS WITH INTEGRATION OF THE PROVIRUS INTO THE HOST CHROMOSOMES
3. CHARACTERISATION OF CONSTITUTIVE IL2 EXPRESSION AND RELEASE BY TRANSDUCED TUMOUR CELLS
4. INJECTION OF CHARACTERISED IL2-TRANSDUCED TUMOUR CELLS INTO THE HOST
5. AMPLIFICATION OR GENERATION OF NK, LAK AND CTL EFFECTORS
6. REDUCED OR ABROGATED TUMORIGENICITY
7. GENERATION OF ANTI-TUMOUR MEMORY

released by the injected cells may elicit an anti-tumour immune response possibly contributed by the generation of cytotoxic cells specifically directed against the tumour, gains further strength by the recent evidence that in the peripheral blood of melanoma patients specific anti-tumour cytotoxic lymphocyte precursors have been described (Coulie *et al.*, 1992). It is conceivable that these cytotoxic cells may be amplified *in vivo* following repeated boosting with IL2-gene transduced tumour cells. The possibility that this may occur gains strength by the evidence that in the P815 mastocytoma-bearing mice the number of tumour-specific CTL could be increased following immunisation with IL2-producing tumour cells (Ley *et al.*, 1991). Hopefully, these cytotoxic effectors would then recirculate and reach the sites of residual disease, and, potentially, confer an anti-tumour immunological memory.

Necessarily, the early clinical applications will have to be through phase I studies aimed at assessing the feasibility and safety of this new approach. It is, however, likely that this innovative therapeutic modality will find a more suitable and potentially fruitful use in the setting of patients with limited or minimal residual disease, where the tumour load is considerably lower and the immune system of the host less compromised. This latter aspect is further supported by studies in acute leukaemia, in which a marked defect of the killing machinery against autologous blasts has been documented at diagnosis and, to a further extent, at relapse, while in the remission phase of the disease there is often a restoration of the autologous killing capacity (Foa *et al.*, 1991*c*). In view of the potentially lowest tumour burden and of the presence of circulating cytotoxic effectors in patients who have undergone an autografting procedure (Reittie *et al.*, 1989; Higuchi *et al.*, 1989), this clinical setting conceptually represents an optimal scenario for future protocols of gene-mediated vaccinations. Finally, since tumour specific antigens are being progressively recognised through more sophisticated cloning technologies (Van Der Bruggen *et al.*, 1991; Chen *et al.*, 1992), it is realistic to speculate that in the near future more powerful and specific vectors containing both a given tumour antigen and the IL2 gene may be constructed and applied to the management of cancer patients.

Work supported by Istituto Superiore di Sanita', Roma (Italy-USA Program on 'Therapy of Neoplasias') (R.F.), by Consiglio Nazionale delle Ricerche, Roma ('Applicazioni Cliniche della Ricerca Oncologica') (R.F.), by American-Italian Foundation for Cancer Research (R.F.), New York and by Schultz Foundation (B.G.). A.G. was partly supported by USL VIII, Torino. We wish to thank Prof. G. Forni (Torino) for helpful discussion.

### References

ADLER, A., CHERVENICK, P.A., WHITESIDE, T.L., LOTZOVA, E. & HERBERMAN, R.B. (1988). Interleukin 2 induction of lymphokine-activated killer (LAK) activity in the peripheral blood and bone marrow of acute leukemia patients. I. Feasibility of LAK generation in adult patients with active disease and in remission. *Blood*, **71**, 709–716.

ANICHINI, A., FOSSATI, G. & PARMIANI, G. (1985). Clonal analysis of cytotoxic T-lymphocyte response to autologous human metastatic melanoma. *Int. J. Cancer*, **35**, 683–689.

ASHER, A.L., MULE', J.J., KASID, A., RESTIFO, N.P., SALO, J.C., REICHERT, C.M., JAFFE, G., FENDLY, B., KRIEGLER, M. & ROSENBERG, S.A. (1991). Murine tumor cells transduced with the gene for tumor necrosis factor-alpha. *J. Immunol.*, **146**, 3227–3234.

BLAISE, D., OLIVE, D., STOPPA, A.M., VIENS, P., POURREAU, C., LOPEZ, M., ATTAL, M., JASMIN, C., MONGES, G., MAWAS, C., MANNONI, P., PALMER, P., FRANKS, C., PHILIP, T. & MARANINCHI, D. (1990). Hematologic and immunologic effects of the systemic administration of recombinant interleukin-2 after autologous bone marrow transplantation. *Blood*, **76**, 1092–1097.

BLANKENSTEIN, T., QIN, Z., UBERLA, K., MULLER, W., ROSEN, H., VOLK, H.-D. & DIAMANTSTEIN, T. (1991). Tumor suppression after tumor cell-targeted tumor necrosis factor α gene transfer. *J. Exp. Med.*, **173**, 1047–1052.

CALIGIURI, M.A., MURRAY, C., SOIFFER, R.J., KLUMPP, T.R., SEIDEN, M., COCHRAN, K., CAMERON, C., ISH, C., BUCHANAN, L., PERILLO, D., SMITH, K. & RITZ, J. (1991). Extended continuous infusion low-dose recombinant interleukin-2 in advanced cancer: prolonged immunomodulation without significant toxicity. *J. Clin. Oncol.*, **9**, 2110–2119.

CHEN, W., PEACE, D.J., ROVIRA, D.K., YOU, S.-G. & CHEEVER, M.A. (1992). T-cell immunity to the joining region of p210BCR-ABL protein. *Proc. Natl Acad. Sci. USA*, **89**, 1468–1472.

COLOMBO, M.P., FERRARI, G., STOPPACCIARO, A., PARENZA, M., RODOLFO, M., MAVILIO, F. & PARMIANI, G. (1991). Granulocyte colony-stimulating factor gene transfer suppresses tumorigenicity of a murine adenocarcinoma *in vivo*. *J. Exp. Med.*, **173**, 889–897.

CORTESINA, G., DE STEFANI, A., GIOVARELLI, M., BARIOGLIO, M.G., CAVALLO, G.P., JEMMA, C., & FORNI, G. (1988). Treatment of recurrent squamous cell carcinoma of the head and neck with low doses of interleukin-2 injected perilymphatically. *Cancer*, **62**, 2482–2485.

COULIE, P.G., SOMVILLE, M., LEHMANN, F., HAINAUT, P., BRASSEUR, F., DEVOS, R. & BOON, T. (1992). Precursor frequency analysis of human cytolytic T lymphocytes directed against autologous melanoma cells. *Int. J. Cancer*, **50**, 289–297.

DAWSON, M.M., JOHNSTON, D., TAYLOR, G.M. & MOORE, M. (1986). Lymphokine activated killing of fresh human leukaemias. *Leuk. Res.*, **10**, 683–688.

DE VRIES, J.E. & SPITS, H. (1984). Cloned human cytotoxic T lymphocyte (CTL) lines reactive with autologous melanoma cells. *J. Immunol.*, **132**, 510–519.

EGLITIS, M.A. & ANDERSON, W.F. (1988). Retroviral vectors for the introduction of genes into mammalian cells. *BioTechniques*, **6**, 608–614.

ERARD, F., CORTHESY, P., NABHOLZ, M., LOWENTHAL, J.W., ZAECH, P., PLAETINCK, G. & MACDONALD, H.R. (1985). Interleukin 2 is both necessary and sufficient for the growth and differentiation of lectin-stimulated cytolytic T lymphocyte precursors. *J. Immunol.*, **134**, 1644–1652.

FAVROT, M.C., COMBARET, V., NEGRIER, S., PHILIP, I., THIESSE, P., FREYDEL, C., BIJMANN, J.T., FRANKS, C.R., MERCATELLO, A. & PHILIP, T. (1990). Functional and immunophenotypic modifications induced by interleukin-2 did not predict response to therapy in patients with renal cell carcinoma. *J. Biol. Response Mod.*, **9**, 167–177.

FEARON, E.R., PARDOLL, D.M., ITAYA, T., GOLUMBEK, P., LEVITSKY, H.I., SIMONS, J.W., KARASUYAMA, H., VOGELSTEIN, B. & FROST, P. (1990). Interleukin-2 production by tumor cells bypasses T helper function in the generation of an antitumor response. *Cell*, **60**, 397–403.

FIERRO, M.T., XIN-SHENG, L., LUSSO, P., BONFERRONI, M., MATERA, L., CESANO, A., LISTA, P., ARIONE, R., FORNI, G. & FOA, R. (1988). *In vitro* and *in vivo* susceptibility of human leukemic cells to lymphokine activated killer activity. *Leukemia*, **2**, 50–54.

FOA, R., MELONI, G., TOSTI, S., FIERRO, M.T., GAVOSTO, F. & MANDELLI, F. (1989). Recombinant IL2 in the treatment of acute leukemia: a pilot study. *Blood*, **74** (suppl 1), 357a.

FOA, R., CARETTO, P., FIERRO, M.T., BONFERRONI, M., CARDONA, S., GUARINI, A., LISTA, P., PEGORARO, L., MANDELLI, F., FORNI, G. & GAVOSTO, F. (1990*a*). Interleukin 2 does not promote the *in vitro* and *in vivo* proliferation and growth of human acute leukaemia cells of myeloid and lymphoid origin. *Br. J. Haematol.*, **75**, 34–40.

FOA, R., FIERRO, M.T., TOSTI, S., MELONI, G., GAVOSTO, F. & MANDELLI, F. (1990*b*). Induction and persistence of complete remission in a resistant acute myeloid leukemia patient following treatment with recombinant interleukin 2. *Leuk. Lymph.*, **1**, 113–117.

FOA, R., MELONI, G., TOSTI, S., NOVARINO, A., FENU, S., GAVOSTO, F. & MANDELLI, F. (1991*a*). Treatment of acute myeloid leukaemia patients with recombinant interleukin 2: a pilot study. *Br. J. Haematol.*, **77**, 491–496.

FOA, R., GUARINI, A., GILLIO TOS, A., CARDONA, S., FIERRO, M.T., MELONI, G., TOSTI, S., MANDELLI, F. & GAVOSTO, F. (1991*b*). Peripheral blood and bone marrow immunophenotypic and functional modifications induced in acute leukemia patients treated with Interleukin 2: evidence of *in vivo* lymphokine activated killer cell generation. *Cancer Res.*, **51**, 964-968.

FOA, R., FIERRO, M.T., CESANO, A., GUARINI, A., BONFERRONI, M., RASPADORI, D., MINIERO, R., LAURIA, F. & GAVOSTO, F. (1991*c*). Defective lymphokine-activated killer cell generation and activity in acute leukemia patients with active disease. *Blood*, **78**, 1041-1046.

FORNI, G., GIOVARELLI, M. & SANTONI, A. (1985). Lymphokine-activated tumor inhibition *in vivo*. The local administration of interleukin 2 triggers nonreactive lymphocytes from tumor-bearing mice to inhibit tumor growth. *J. Immunol.*, **134**, 1305-1311.

FORNI, G., FUJIWARA, H., MARTINO, F., HAMAOKA, T., JEMMA, C., CARETTO, P. & GIOVARELLI, M. (1988). Helper strategy in tumor immunology: expansion of helper lymphocytes and utilization of helper lymphokines for experimental and clinical immunotherapy. *Cancer Met. Rev.*, **7**, 289-295.

GANSBACHER, B., BANNERJI, R., DANIELS, B., ZIER, K., CRONIN, K. & GILBOA, E. (1990*a*). Retroviral vector-mediated γ-interferon gene transfer into tumor cells generates potent and long lasting antitumor immunity. *Cancer Res.*, **50**, 7820-7825.

GANSBACHER, B., ZIER, K., DANIELS, B., CRONIN, K., BANNERJI, R. & GILBOA, E. (1990*b*). Interleukin 2 gene transfer into tumor cells abrogates tumorigenicity and induces protective immunity. *J. Exp. Med.*, **172**, 1217-1224.

GANSBACHER, B., GUARINI, A., GILBOA, E. & GOLDE, D. (1991). Lymphoma regression induced by gene transfer mediated localized IL-2 secretion. *Blood*, **78**, Suppl 1, 384a.

GANSBACHER, B., ZIER, K., HANTZOPOULOS, A., HOUGHTON, A., GILBOA, E. & GOLDE, D. (1992). Retroviral gene transfer induces constitutive expression of IL-2 and IFN-gamma in irradiated human melanoma cells. *Blood*, (in press).

GASTL, G., FINSTAD, C.L., GUARINI, A., BOSL, G., GILBOA, E., BANDER, N.H. & GANSBACHER, B. (1992). Retroviral vector-mediated lymphokine gene transfer into human renal cancer cells. *Cancer Res.*, (in press).

GILBOA, E., EGLITIS, M.A., KANTOFF, P.W. & ANDERSON, W.F. (1986). Transfer and expression of cloned genes using retroviral vectors. *BioTechniques*, **4**, 504-512.

GOLDSTEIN, D. & LASZLO, J. (1986). Interferon therapy in cancer: from imaginon to interferon. *Cancer Res.*, **46**, 4315-4329.

GOLUMBEK, P.T., LAZENBY, A.J., LEVITSKY, H.I., JAFFEE, L.M., KARASUYAMA, H., BAKER, M. & PARDOLL, D.M. (1991). Treatment of established renal cancer by tumor cells engineered to secrete interleukin-4. *Science*, **254**, 713-716.

GOTTLIEB, D.J., PRENTICE, H.G., HESLOP, H.E., BELLO-FERNANDEZ, C., BIANCHI, A.C., GALAZKA, A.R. & BRENNER, M.K. (1989). Effects of recombinant interleukin-2 administration on cytotoxic function following high-dose chemo-radiotherapy for hematological malignancies. *Blood*, **74**, 2335-2342.

GRIMM, E.A., MAZUMDER, A., ZHANG, H.Z. & ROSENBERG, S.A. (1982). Lymphokine-activated killer cell phenomenon. Lysis of natural killer-resistant fresh solid tumor cells by interleukin 2-activated autologous human peripheral blood lymphocytes. *J. Exp. Med.*, **155**, 1823-1841.

HANK, J.A., KOHLER, P.C., WEIL-HILLMAN, G., ROSENTHAL, N., MOORE, K.H., STORER, B., MINKOFF, D., BRADSHAW, J., BECHHOFER, R. & SONDEL, P.M. (1988). *In vivo* induction of the lymphokine-activated killer phenomenon: interleukin 2-dependent human non-major histocompatibility complex-restricted cytotoxicity generated *in vivo* during administration of human recombinant interleukin 2. *Cancer Res.*, **48**, 1965-1971.

HESLOP, H.E., GOTTLIEB, D.J., BIANCHI, A.C.M., MEAGER, A., PRENTICE, H.G., MEHTA, A.B., HOFFBRAND, A.V. & BRENNER, M.K. (1989). *In vivo* induction of gamma interferon and tumor necrosis factor by interleukin-2 infusion following intensive chemotherapy or autologous marrow transplantation. *Blood*, **74**, 1374-1380.

HIGUCHI, C.M., THOMPSON, J.A., COX, T., LINDGREN, C.G., BUCKNER, D.C. & FEFER, A. (1989). Lymphokine-activated killer function following autologous bone marrow transplantation for refractory hematological malignancies. *Cancer Res.*, **49**, 5509-5513.

HIGUCHI, C.M., THOMPSON, J.A., PETERSEN, F.B., BUCKNER, C.D. & FEFER, A. (1991). Toxicity and immunomodulatory effects of interleukin-2 after autologous bone marrow transplantation for hematologic malignancies. *Blood*, **77**, 2561-2568.

HOCK, H., DORSCH, M., DIAMANTSTEIN, T. & BLANCKENSTEIN, T. (1991). Interleukin-7 induces CD4+ T cell-dependent tumor rejection. *J. Exp. Med.*, **174**, 1291-1298.

LAFRANIERE, R. & ROSENBERG, S.A. (1985). Successful immunotherapy of murine experimental hepatic metastases with lymphokine-activated killer cells and recombinant interleukin 2. *Cancer Res.*, **45**, 3735-3741.

LEY, V., LANGLADE-DEMOYEN, P., KOURILSKY, P. & LARSSON-SCIARD, E-L. (1991). Interleukin 2-dependent activation of tumor-specific cytotoxic T lymphocytes *in vivo*. *Eur. J. Immunol.*, **21**, 851-854.

LIM, S.H., NEWLAND, A.C., KELSEY, S., BELL, A., OFFERMAN, E., RIST, C., GOZZARD, D., BAREFORD, D., SMITH, M.P. & GOLDSTONE, A.H. (1992). Continuous intravenous infusion of high-dose recombinant interleukin-2 for acute myeloid leukaemia - a phase II study. *Cancer Immunol. Immunother.*, **34**, 337-342.

LISTA, P., FIERRO, M.T., XIN-SHENG, L., BONFERRONI, M., BRIZZI, M.F., PORCU, P.L., PEGORARO, L. & FOA, R. (1989). Lymphokine-activated killer (LAK) cells inhibit the clonogenic growth of human leukaemic stem cells. *Eur. J. Haematol.*, **42**, 425-430.

LOTZOVA, E., SAVARY, C.A. & HERBERMAN, R.B. (1987). Inhibition of clonogenic growth of fresh leukemia cells by unstimulated and IL-2 stimulated NK cells of normal donors. *Leukemia Res.*, **15**, 245-254.

MARANINCHI, D., BLAISE, D., VIENS, P., BRANDELY, M., OLIVE, D., LOPEZ, M., SAINTY, D., MARIT, G., STOPPA, A.M., REIFFERS, J., GRATECOS, N., BERTAU-PEREZ, P., MANNONI, P., MAWAS, C., HERCEND, T., SEBAHOUN, G. & CARCASSONNE, Y. (1991). High-dose recombinant interleukin-2 and acute myeloid leukemias in relapse. *Blood*, **78**, 2182-2187.

MCLACHLIN, J.R., CORNETTA, K., EGLITIS, M.A. & ANDERSON, F. (1990). Retroviral-mediated gene transfer. *Progr. Nucl. Acid Res. Mol. Biol.*, **38**, 91-135.

MELONI, G., FOA, R., TOSTI, S., VIGNETTI, M., MANCINI, F., GUARINI, A., MARCHIS, D., GAVOSTO, F. & MANDELLI, F. (1992). Autologous bone marrow transplantation followed by interleukin 2 in children with advanced leukemia: a pilot study. *Leukemia*, (in press).

MORGAN, D.A., RUSCETTI, F.W. & GALLO, R.C. (1976). Selective *in vitro* growth of T lymphocytes from normal human bone marrows. *Science*, **193**, 1007-1008.

MULE', J.J., SHU, S., SCHWARZ, S.L. & ROSENBERG, S.A. (1984). Successful adoptive immunotherapy of established pulmonary metastases with LAK cells and recombinant IL-2. *Science*, **255**, 1487-1489.

ORTALDO, J.R., MASON, A.T., GERARD, J.P., HENDERSON, L.E., FARRAR, W., HOPKINS, R.F. III, HERBERMAN, R.B. & RABIN, H. (1984). Effects of natural and recombinant IL-2 on regulation of IFN production and natural killer activity: lack of involvement of the Tac antigen for these immunoregulatory effects. *J. Immunol.*, **133**, 779-783.

OSHIMI, K., OSHIMI, Y., AKUTSU, M., TAKEI, Y., SAITO, H., OKADA, M. & MIZOGUCHI, H. (1986). Cytotoxicity of interleukin 2 activated lymphocytes for leukemia and lymphoma cells. *Blood*, **68**, 938-948.

PARKINSON, D.R., ABRAMS, J.S., WIERNIK, P.H., RAYNER, A.A., MARGOLIN, K.A. VAN ECHO, D.A., SZNOL, M., DUTCHER, J.P., ARONSON, F.R., DOROSHOW, J.H. ATKINS, M.B. & HAWKINS, M.J. (1990). Interleukin-2 therapy in patients with metastatic malignant melanoma: A phase II study. *J. Clin. Oncol.*, **8**, 1650-1656.

PORGADOR, A., TZEHOVAL, E., KATZ, A., VADAI, E., REVEL, M., FELDMAN, M. & EISENBACH, L. (1992). Interleukin 6 gene transfection into Lewis lung carcinoma tumor cells suppresses the malignant phenotype and confers immunotherapeutic competence against parental metastatic cells. *Cancer Res.*, **52**, 3679-3686.

REITTIE, J., GOOTLIEB, D., HESLOP, H., LEGER, O., HAZLERHURST, G., DREXLER, H., HOFFBRAND, A., PRENTICE, H. & BRENNER, M. (1989). Endogenously generated activated killer cells circulate after autologous and allogeneic marrow transplantation. *Blood*, **73**, 1351-1358.

ROSENBERG, S.A., LOTZE, M.T., MUUL, L.M., LEITMAN, S., CHANG, A.E., ETTINGHAUSEN, S.E., MATORY, Y.L., SKIBBER, J.M., SHILONI, E., VETTO, J.T., SEIPP, C.A., SIMPSON, C. & REICHERT, C.M. (1985). Observations on the systemic administration of autologous lymphokine-activated killer cells and recombinant interleukin-2 to patients with metastatic cancer. *N. Engl. J. Med.*, **313**, 1485-1492.

ROSENBERG, S.A., SPIESS, P. & LAFRANIERE, R. (1986). A new approach to the adoptive immunotherapy of cancer with tumor-infiltrating lymphocytes. *Science*, **223**, 1218-1221.

ROSENBERG, S.A., LOTZE, M.T., MUUL, L.M., CHANG, A.E., AVIS, F.P., LEITMAN, S., LINEHAN, W.M., ROBERTSON, C.N., LEE, R.E., RUBIN, J.T., SEIPP, C.A., SIMPSON, C.G. & WHITE, D.E. (1987). A progress report on the treatment of 157 patients with advanced cancer using lymphokine-activated killer cells and interleukin-2 or high-dose interleukin-2 alone. *N. Engl. J. Med.*, **316**, 889–897.

ROSENBERG, S.A., PACKARD, B.S., AEBERSOLD, P.M., SOLOMON, D., TOPALIAN, S.L., TOY, S.T., SIMON, P., LOTZE, M.T., YANG, J.C., SEIPP, C.A., SIMPSON, C., CARTER, C., BOCK, S., SCHWARTZENTRUBER, D., WEI, J.P. & WHITE, D.E. (1988). Immunotherapy of patients with metastatic melanoma using tumor infiltrating lymphocytes and interleukin-2: preliminary report. *N. Engl. J. Med.*, **319**, 1676–1680.

ROSENBERG, S.A., LOTZE, M.T., YANG, J.C., AEBERSOLD, P.M., LINEHAN, W.M., SEIPP, C.A. & WHITE, D.E. (1989). Experience with the use of high-dose interleukin-2 in the treatment of 652 cancer patients. *Ann. Surg.*, **210**, 474–484.

ROSENBERG, S.A. (1991). Immunotherapy and gene therapy of cancer. *Cancer Res.*, **51** (suppl.), 5074s–5079s.

RUSSELL, S.J., ECCLES, S.A., FLEMMING, C.L., JOHNSON, C.A. & COLLINS, M.K. (1991). Decreased tumorigenicity of a transplantable rat sarcoma following transfer and expression of an IL-2 cDNA. *Int. J. Cancer*, **47**, 244–251.

SMITH, K.A. (1988). Interleukin-2: inception, impact, and implications. *Science*, **240**, 1169–1176.

SOIFFER, R.J., MURRAY, C., COCHRAN, K., CAMERON, C., WANG, E., SCHOW, P.W., DALEY, J.F. & RITZ, J. (1992). Clinical and immunologic effects of prolonged infusion of low-dose recombinant interleukin-2 after autologous and T-cell-depleted allogeneic bone marrow transplantation. *J. Clin. Oncol.*, **79**, 517–526.

SONDEL, P.M., KOHLER, P.C., HANK, J.A., MOORE, K.H., ROSENTHAL, N.S., SOSMAN, J.A., BECHHOFER, R. & STORER, B. (1988). Clinical and immunological effect of recombinant interleukin 2 given by repetitive cycles to patients with cancer. *Cancer Res.*, **48**, 2561–2567.

TEPPER, R.I., PATTENGALE, P.K. & LEDER, P. (1989). Murine interleukin-4 displays potent anti-tumor activity *in vivo*. *Cell*, **57**, 503–512.

THOMPSON, J.A., SHULMAN, K.L., BENYUNES, M.C., LINDGREN, C.G., COLLINS, C., LANGE, P.H., BUSH, W.H. Jr, BENZ, L.A. & FEFER, A. (1992). Prolonged continuous intravenous infusion interleukin-2 and lymphokine-activated killer cell therapy for metastatic renal cell carcinoma. *J. Clin. Oncol.*, **10**, 960–968.

VAN DER BRUGGEN, P., TRAVERSARI, C., CHOMEZ, P., LURQUIN, C., DE PLAEN, E., VAN DEN EYNDE, B., KNUTH, A. & BOON, T. (1991). A gene encoding an antigen recognized by cytolytic T lymphocytes on a human melanoma. *Science*, **254**, 1643–1647.

WATANABE, Y., KURIBAYASHI, K., MIYATAKE, S., NISHIHARA, K., NAKAYAMA, E-I., TANIYAMA, T. & SAKATA, T-A. (1989). Exogenous expression of mouse interferon-γ cDNA in mouse neuroblastoma C1300 cells results in reduced tumorigenicity by augmented anti-tumor immunity. *Proc. Natl Acad. Sci. USA*, **86**, 9456–9460.

WEST, W.H., TAUER, K.W., YANNELLI, J.R., MARSHALL, G.D., ORR, D.W., THURMAN, G.B. & OLDHAM, R.K. (1987). Constant-infusion recombinant interleukin-2 in adoptive immunotherapy of advanced cancer. *N. Engl. J. Med.*, **316**, 898–905.